# Congenital heart disease and Down syndrome: various aspects of a confirmed association

**DOI:** 10.5830/CVJA-2016-019

**Published:** 2016

**Authors:** Benhaourech, Sanaa, Drighil, Abdenasser, El Hammiri Ayoub

**Affiliations:** Cardiology Department, University Hospital Ibn Rochd,Casablanca, Morocco; Cardiology Department, University Hospital Ibn Rochd,Casablanca, Morocco; Cardiology Department, University Hospital Ibn Rochd,Casablanca, Morocco

**Keywords:** Down syndrome, congenital heart disease, epidemiology, therapeutic

## Abstract

**Background:**

Congenital heart disease (CHD) is frequently described in patients with Down syndrome (DS) and is the main cause of death in this population during the first two years of life. The spectrum of CHD patterns in DS varies widely worldwide; this variation could be due to sociodemographic, genetic and geographic factors.

**Methods:**

A six-year retrospective, descriptive study was carried out from December 2008 to October 2014, based on the Paediatric Unit CHD registry of Ibn Rochd University Hospital. Clinical, echocardiographic and outcomes data were collected and sorted according to confirmation of the syndrome.

**Results:**

Among 2 156 patients with CHD, 128 were identified with Down syndrome. The genders were equally represented (gender ratio 1) and the median age at diagnosis was 9.5 months (2 days to 16 years). The median age of mothers at delivery was 39 years (16–47). Of the 186 CHD lesions reported, the most common was atrioventricular septal defect (AVSD, 29%), followed by ventricular septal defect (VSD,21.5%) and atrial septal defect (ASD, 19.9%). The most common associations of CHD were AVSD + ASD (10%) and VSD + ASD (7.8%). Surgery was the most common modality of treatment (54.3%). The overall mortality rate was 14.1%.

**Conclusion:**

Our study confirmed that the profile and type of CHD in DS in the Moroccan setting exhibited slight differences in the distribution of these CHDs compared with European neighbours and other Western countries. Further studies are needed to determine which variables have an impact on these differences.

## Background

Down syndrome (DS), which is caused by trisomy on chromosome 21, is by far the most common and best known chromosomal disorder in humans and the most common cause of intellectual disability.^1^-^3^ This trisomy gives rise to multiple complications as part of the syndrome. Congenital heart disease(CHD) is the leading cause of mortality and morbidity duringthe first two years of life in the DS population,^1^,^4^ and 40 to 63.5% of DS patients have CHD.^4^-^6^

It has been suggested that the profile and type of these CHDs are variable according to the different geographical areas around the world.^7^,^8^ Recent studies in Norway also suggest a seasonal variation in the occurrence of DS and birth defects, and provide indirect evidence of the causal role of environmental factors,since genetic factors do not exhibit seasonality.^9^ Because Morocco is bordered by European countries, it has been suggested that a combination of local factors and regional proximity could play a significant role in the CHD profile in the DS population.

However, in a given context, it is important to be familiar with the incidence and anatomical characteristics of CHD in DS, as well as the associated complications and causes of morbidity and mortality, in order to apply preventative measures and to improve the patient’s quality of life. In addition, because the type of CHD and the timing of repair affect the prognosis,timely treatment of cardiac abnormalities is crucial for optimal survival.^10^ The lack of reliable data from African countries is a limiting factor in addressing the issue of geographical variations around the world.

The reported rates of different features of CHDs in DS patients between countries in close proximity are quite similar, such as the USA and Mexico or other Latin-Americans countries.^3^,^4^,^8^,^11^ This could be explained by regional proximity, which may have a greater effect in instances of geographical areas with longstanding populations, as is the case in the Mediterranean area.

Morocco is a North African country that has historical links with European populations in the Mediterranean area, but also with those of Africa in the south. This study sought to determine the prevalence and profile of CHD in DS in the Moroccan context and to compare this with the international literature.

## Methods

This retrospective, descriptive, monocentric study was based on the Paediatric Unit CHD registry of Ibn Rochd University Hospital. All CHD-affected patients diagnosed with DS (with or without chromosomal studies) during the period from December 2008 to November 2014 were included in the study.

Phenotypic clinical features matching with DS recorded in the medical charts were as follows: mongoloid facies, protruding tongue, transverse single palmar crease, brachycephaly,depressed nasal bridge, small, low-set ears, and upward-slanted eyes with epicanthic fold, short neck and hypotonia. General characteristics such as gender, age of diagnosis and mother’s age at delivery were also recorded.

The examination protocol during echocardiographic assessment was as follows: subxiphoid imaging followed by a segmental approach for description of the major cardiovascular structures in sequence, with the image apex at the bottom of the video. We recorded all videos of the examinations and all cases underwentdetailed review by the paediatric cardiologist (AD) to classify CHD according to standard nomenclature used by the Society of Thoracic Surgery congenital heart surgery database (Table 1).^12^

**Table 1:** Types of congenital heart defects in patients with Down syndrome

**	*CHD feature, n (%)*
*Type of CHD*	*(n = 186)*
Atrioventricular septal defect (AVSD)	54 (29)
Complete AVSD	46/54 (85)
• Rastelli type A	12/54 (26)
• Rastelli type B	0
• Rastelli type C	34/54 (74)
• Balanced	45/54 (98)
• Unbalanced	1/54 (2)
Partial AVSD	1 (1.9)
Intermediate AVSD	7 (12.9)
Ventricular septal defect (VSD)	40 (21.5)
Perimembranous	28/40 (70)
Subpulmonary	4/40 (10)
Muscular	4/40 (10)
Inlet	4/40 (10)
Atrial septal defect (ASD)	37 (19.9)
Ostium secundum	35/37 (95)
Sinus venosus	2/37 (5)
Patent ductus arteriosus (PDA)	31 (16.7)
Tetralogy of Fallot (FT)	10 (5.4)
Valvular disease	6 (3.2)
Mitral valvular insufficiency	5/6 (83)
Aorticvalvular insufficiency	1/6 (17)
Other	8 (4.3)
Left superior vena cava	3/8 (37)
Pulmonary stenosis	2/8 (25)
Coartaction of the aorta	1/8 (12)
PAPVC	1/8 (12)
Double outlet right ventricle	1/8 (12)

PAPVC: partial anomalous pulmonary venous connection.

## Statistical analysis

Data are reported as mean ± SD or median with intervals. We used the t-test for paired data. The SPSS 20 (IBM SPSS Statistics version 20 x86 Multiple languages) and Origin statistical packages were used.

## Results

**Fig. 1:**
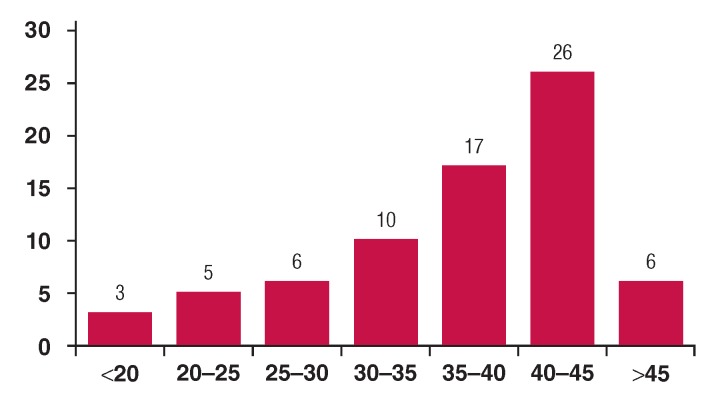
Mothers’ ages of patients with Down syndrome at delivery.

We recorded 2 156 patients with CHD and of these, 128 (6%) had DS. The median age of patients at diagnosis was 9.5 months (2 days to 16 years), with 40.4% of patients diagnosed before six months of age. The male-to-female ratio was 1:1. The median age of mothers at delivery was 39 years (16–47) (Fig. 1) and the consanguinity rate was 22%. The distribution of different types of CHD is shown in Table 1. Overall, 186 CHDs were diagnosed in our study population; 75 patients (58.6%) had a single cardiac lesion versus 56 (41.4%) with multiple cardiac lesions.

When considering individual CHDs, atrioventricular septal defect (AVSD, 29.9%) was the most common cardiac abnormality. Among these AVSDs, the complete form was the most frequent type, found in cases 46 cases (85.2%) and, of those complete forms of AVSD, Rastelli class C AVSD was found to be the most frequent and was reported in 34 cases (74%).

Ventricular septal defect (VSD) was the second most common cardiac abnormality (21.5%). Perimembranous VSDs were reported in 28 cases (70%) and other VSD forms were reported in 16 cases (30%).

Atrial septal defect (ASD, 19.9%) was the third most common single lesion. Most of these ASDs were of ostium secundum form (95%), while the other forms (sinus venosus) were noted in 5%. Patent ductus arteriosus (PDA) was diagnosed in 16.57% and tetralogy of Fallot (TOF) in 5%.

The most frequent associations of CHD were AVSD + ASD (9.3%), VSD + ASD (6.2%) and VSD + PDA (5.5%). Pulmonary arterial hypertension (PAH) was seen in 68 patients (53%) and Eisenmeiger syndrome in four patients (3.1%).

**Fig. 2:**
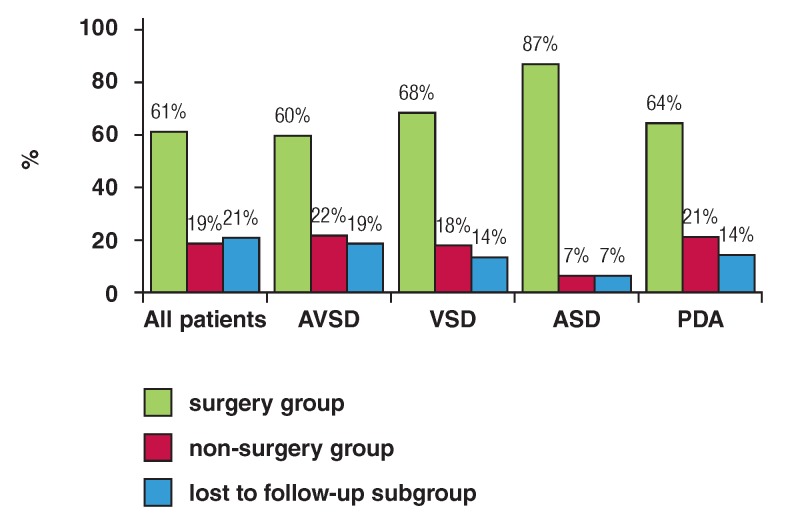
Rates of surgical procedures for the whole group of patients with Down syndrome and congenital heart disease and for the most frequent congenital heart defects in these patients. AVSD: atrioventricular septal defect; VSD: ventricular septal defect. ASD: atrial septal defect; PDA: patent ductus arteriosus.

Surgery was indicated in 54% (69 patients) but for many reasons (no agreement from the parents, lack of financial resources, etc), a surgical procedure was done in only 42 patients. In those surgically treated patients, 87% presented with ASD, and 68, 64 and 60% with VSD, PDA and AVSD, respectively (Fig. 2). In addition, 23 and 15% of patients remained either under medical treatment or observation, respectively.

The mortality rate observed in our population was 14.1%, and another 29.7% were lost to follow up (Table 2). For thosepatients with an indication for surgery who were operated on, 79% survived. On the other hand, for the patients having a surgical indication but were not operated on, only 31% survived.

**Table 2:** Fate of patients with Down syndrome and congenital heart disease

**	*Number of patients (%)*
*Patients with DS and CHD*	*(n = 128)*
• Dead	18 (14.1)
• Alive	72 (56.3)
• Lost to follow up	38 (29.7)
Patients with an indication for surgery	69/128 (54)
Patients operated on	42/69 (61)
• Dead	8/42 (19)
• Alive	33/42 (79)
• Lost to follow up	1/42 (2)
Patients not operated on	13/69 (19)
• Dead	9/13 (69)
• Alive	4/13 (31)
• Lost to follow up	14/69 (20)
Patients with no indication for surgery	59/128 (46)
• Dead	1/59 (2)
• Alive	35/59 (59)
• Lost to follow up	23/59 (39)

## Discussion

CHD in DS is reported to be as high as 40 to 63% and is a major cause of morbidity and early mortality in these patients.^4^,^6^,^8^,^13^ It has been suggested that when characterising the profiles and types of CHD in DS, the dominant lesions observed are variable according to the different geographical areas around the world.^7^,^8^ Hence, for a given country, to know the profile and characteristics of CHD in DS is of great importance, first to improve survival by timely treatment of cardiac anomalies,^10^ and second to apply appropriate preventative measures.

In this study, we sought to determine the distribution of CHD in DS in the Moroccan setting. As our institution is a referral centre for approximately one-quarter of the population of our country, the results observed in this study may reflect the national trends of CHD in DS.

AVSD was the most common cardiac abnormality and VSD the second most common abnormality. Together, AVSD and VSD cases represented 50% of CHDs in our setting. ASD, isolated PDA and tetralogy of Fallot were recorded at rates of 19.9, 16 and 5%, respectively.

DS is the most common autosomal abnormality. The frequency is about one case in 600 live births.^1^-^3^ This syndrome, which is by far the most common and best known chromosomal disorder in humans, is characterised by intellectual disability, dysmorphic facial features and other distinctive phenotypic traits.^1^-^4^,^6^

DS is primarily caused by trisomy of chromosome 21, which is the most common trisomy among live births. In 94% of patients with DS, full trisomy 21 is the cause; mosaicism (2.4%) and translocations (3.3%) account for the remaining cases.^14^,^15^ Approximately 75% of the unbalanced translocations are de novo, and approximately 25% result from familial translocation.^14^

Two different hypotheses have been proposed to explain the mechanism of gene action in DS: developmental instability (i.e. loss of chromosomal balance) and the so-called gene-dosage effect.^15^ According to the gene-dosage effect hypothesis, the genes located on chromosome 21 have been overexpressed in cells and tissues of DS patients, and this contributes to the phenotypic abnormalities.^16^

There has been much interest in trying to identify the exact location of CHD susceptibility genes. Although trisomy 21 is a risk factor for CHD, it is not a sufficient requirement (about 40–60% of people with trisomy 21 do not have CHD).

Molecular mapping studies suggested the presence of a ‘critical region’ that is responsible for the various CHD phenotypes, and narrowed the region to D21S3 (defined by VSDs) through to PFKL (defined by tetralogy of Fallot), containing 39 human genes and 25 predicted genes. One of these genes, DSCAM, is known to mediate cell–cell adhesion, thought to be essential to the process of cellular adhesion and fusion of endocardial cushions. It is speculated that the overexpression of DSCAM can lead to a disturbance of normal epithelial– mesenchymal transformation and/or mesenchyme cell migration or proliferation, thus resulting in an increase in the adhesive property of the cushion fibroblasts, leading to the various heart defects.^17^

As observed during this study, median age of the mothers at delivery was 39 years (16–47). The occurrence of DS is strongly dependent on maternal age, and advanced maternal age remains the only well-documented risk factor for maternal meiotic non-disjunction. With a maternal age of 45 years, the risk is one in 30 to 50 live births.^18^ However, understanding of the basic mechanism behind the maternal age effect is lacking. Some studies also suggest a role for consanguinity^19^ (Fig. 1).

In our study, the most common lesion was AVSD (29%), followed by VSD (21.5%) and ASD (19.9%). The most common associations of CHD were AVSD + ASD (10%) and VSD + ASD (7.8%) (Table 1).

In the international literature, the most common CHDs in DS from reports from western European countries and the USA are the following: endocardial cushion defect (43%), which results in AVSD/AV canal defect; VSD (32%); secundum atrial septal defect (10%); tetralogy of Fallot (6%); and isolated PDA (4%). About 30% of patients have several cardiac defects.^3^,^4^,^6^,^13^ However,in Asia, isolated VSDs have been reported to be the most common defect, observed in about 40% of patients,^20^ whereas in most reports from Latin America, the secundum type of ASD is suggested to be the most common lesion.^8^,^11^

This study on CHD in DS in the Moroccan setting exhibited similar results to those of Western countries in terms of major CHDs in DS, but the prevalence rates of ASVD and VSD were lower. This has also been reported by others^1^,^7^,^8^,^20^ and reinforces the findings of a variation in profile and type of CHD in DS in the different geographical areas around the world.

Since our study exhibited similar CHD dominant lesions in DS to those of Morocco’s neighbouring European countries, it suggests that, despite the level of development of the different countries, a combination of factors and regional proximity most likely plays a significant role in such similarities. However,regional proximity alone cannot explain all the differences, as studies in African countries that also have regional proximity with Europe, such as Libya, have exhibited results that globally encompassed the spectrum of CHD in DS seen in Europe but with quite different rates in the different CHDs.^21^ As illustrated by the differences in reported rates of the different CHDs inDS between countries in close proximity, such as the USA and Mexico or other Latin American countries,^3^,^4^,^8^,^11^ it appears that regional proximity may have a greater effect in cases of geographical areas with long-standing populations, as in the Mediterranean area.

About 40.4% of the patients were evaluated during the first six months of life and all had echocardiograms (100%). As reported by others,^22^ age at evaluation of CHD is an important factor for the reduction in mortality and morbidity rates. It has been suggested that when the associated heart abnormalities are leftto- right shunts, the prognosis is more favourable than when there is associated AVSD, which is linked to pulmonary hypertension, a condition in itself related to high mortality rates.^23^

The high mortality rate observed in our study was associated with many factors, including non-consent of the parents for surgery and the fact that a substantial number of cases did not undergo surgery because of insufficient financial resources (Fig. 2). For various reasons, a substantial number of patients were lost to follow up. This high rate probably reflects the difficulties for families and the health system to cope with such diseases in the context of a developing country (Table 2).

## Conclusion

As suggested in the international literature, our study confirmed that the profile and type of CHDs in DS in the Moroccan context exhibited slight differences in the distribution of these CHDs compared with its European neighbours and/or other Western countries. Further studies are needed to determine which variables have an impact on these differences.
